# Apparent digestibility and nutritional composition of *Leucaena leucocephala* (Lam) leaf meal incorporated in the diets of Black Australorp and Potchefstroom Koekoek chicken breeds

**DOI:** 10.1007/s11250-021-02922-w

**Published:** 2021-09-20

**Authors:** Marupine Windy Thamaga, Hilda Kwena Mokoboki, Nthabiseng Amenda Sebola, Khuliso Emmanuel Ravhuhali

**Affiliations:** 1 Department of Agriculture, Rural Development, Land and Environmental Affairs, Private Bag x3001, Mbibane, Dr JS Moroka Municipality, Mpumalanga Province South Africa; 2 Department of Animal Science, School of Agricultural Sciences, Faculty of Natural and Agricultural Sciences, North West University, Mafikeng Campus, Mmabatho, 2735 South Africa; 3 Food Security and Safety Niche Area, Faculty of Natural and Agricultural Sciences, North West University, Mafikeng Campus, Mmabatho, 2735 South Africa; 4 School of Agriculture and Life Science, Department of Agriculture and Animal Health, University of South Africa, Florida Science Campus, Roodepoort, 1710 South Africa

**Keywords:** Black Australorp, Mature leaves, Nutrient intake, Potchefstroom Koekoek, Tender leaves

## Abstract

The objective of the study was to determine the apparent digestibility and nutrient composition of *Leucaena leucocephala* leaf meal (LLM) inclusion in Black Australorp and Potchefstroom Koekoek diets. Tender and mature leaves were separately harvested from 10 individual trees and stored separately for chemical analyses. The leaves were air-dried in a well-ventilated laboratory to constant weight and milled to pass through a 1-mm sieve. A mixture of tender and mature leaves was also collected to produce a bulk leaf meal. The four iso-nitrogenous dietary treatments were 0 (control), 2.5, 5.0 and 7.5% of LLM, respectively. The apparent digestibility of two chicken breeds was also evaluated. The dry matter (DM), neutral detergent fibre (NDF), ether extract (EE), cellulose and hemicellulose of the samples did not differ between tender and mature leaves. Tender leaves had higher (*P* < 0.05) calcium, potassium, magnesium and sodium concentration than mature leaves. Crude protein and mimosine content were significantly (*P* < 0.05) higher in tender than in mature leaves. The inclusion levels of *L. leucocephala* leaf meal affect (*P* < 0.05) acid detergent fibre (ADF), neutral detergent fibre (NDF) and crude protein (CP) digestibility. Crude protein digestibility decreases as the inclusion levels of *L. leucocephala* increase. Both tender and mature *L. leucocephala* leaves have a potential nutritional value that can be used in feedstuff and can be used as a protein supplement for Black Australorp and Potchefstroom Koekoek chicken breeds.

## Introduction

Poultry production plays a vital role in the improvement of the income and food security of the communal poultry producers (Sonaiya 2003). Chickens are a source of protein to the ever-increasing population in developing countries (Dyubele et al. 2010). Farmers especially in rural areas prefer communal indigenous chickens because they are not capital intensive (Muchadeyi et al. 2007). Communal chicken production refers to birds kept under extensive system, scavenging in the free range, have no identified description, multi-purpose and unimproved (Mngonyama 2012). The most common South African breeds kept in communal areas include Potchefstroom Koekoek, Ovambo, Lebowa-Venda and Naked-Neck. The other European breeds that are kept for free range systems (extensive) are New Hampshire, Rhode Island Red and Black Australorp (Mngonyama 2012).

The Potchefstroom Koekoek was bred during the 1950s at the former Potchefstroom Agricultural Research Institute from cross between the White Leghorn, Black Australorp and Bared Plymouth Rock and is recognized as a locally developed breed (FAO 2014). It is categorized as a heavy breed whereas characterized by relative high egg production and adaptability for household production. It is a dual-purpose breed that lays brown-shelled eggs with an average weight of 55.7 g (Ramsey et al. 2001). The meat of the Potchefstroom Koekoek is popular amongst household communities and is favoured like the commercial broiler hybrids (Grobbelaar 2008). The Potchefstroom Koekoek colour form is a sex-related gene that is beneficial for colour sexing in cross-breeding for egg producing types of hens used in medium input production systems. In African countries, the breed is well known for their egg, mothering ability and meat production (Grobbelaar 2008). Tadelle and Fasil (2016) reported that the average body weight of Potchefstroom males is around 3–4 kg and of females is 2.1 kg. The Potchefstroom Koekoek reaches sexual maturity at 18.5 days of age (Grobbelaar et al. 2010).

The most commonly imported chicken breed used in South Africa is Black Australorp. The Australorp chicken was developed as a result of improving the English Orpington in Australia. They were recognized as Black Utility Orpington. The breed was acknowledged as Standard Perfection in 1929. Australorp chickens are black in colour. Males have an average body weight of 3.85 kg and females 2.94 kg (Fourie and Grobbelaar 2003). The breed is large, soft-feathered chicken with glossy black feathers and a lustrous green sheen. The Black Australorp chicken is hardy, docile, good layer, reach early maturity and also a good meat breed. The body weight of large Australorp roosters is between 3.9 and 4.7 kg, while hens weigh between 3.7 and 4.5 kg. The rooster of a bantam has a body weight between 1.8 and 2.3 kg, while hens weigh between 1.7 and 2.2 kg (small-farm-permiculture-and-sustainability-living.com^2012^).

Okitoi et al. (2006) and Mbajiorgu et al. (2011) reported that the productivity of indigenous chickens is very low and mortality is high, emphasizing that appropriate genetic, nutritional and management interventions are required to appreciate their ultimate production potential. Nutritional strategies must be considered to promote and improve the productivity of indigenous chickens which will effectively contribute to poverty alleviation. Several studies revealed that leaves of *Alchornea cordifolia*, *Chromolaena odorata*, *Leucaena leucocephala* and *Moringa oleifera* are important feed resources which are relatively rich in crude protein (25–44%), essential amino acids, minerals, carotenoids and vitamins (Tendonkeng et al. 2008; Olugbemi et al. 2010; Houndonougbo et al. 2012). Amongst those resources, *Leucaena* can play an important role when included in the diets of egg-laying hens (Safwat et al. 2014). *L. leucocephala* leaves contain 23.3% DM of crude protein, 11.4% DM ash, 2573.8 kcal/kg DM metabolizable energy, 0.38% calcium, 2.9% phosphorus and 1.6% tannins (Onibi et al. 2008; Ayssiwede et al. 2011; Garcia et al. 1996). The plant age or stage of maturity also has substantial effect on the plant nutritional composition (Buxton 1996), whereby an increase in age of the plant has always been associated with an increase of cell wall. However, the limited information about the use and chemical composition of *Leucaena* for small-scale farmers might be a constraint to its use (Leketa et al. 2019). Although *Leucaena* has been reported to be highly palatable, the toxic mimosine content poses a challenge to its use in animal feeding (Sastry and Singh 2008). The purpose of this study was to determine the nutritional composition of air-dried *L. leucocephala* leaves and the digestibility of diets *L. leucocephala* meal (LLM) fed to Potchefstroom Koekoek and Black Australorp chicken breeds.

## Materials and methods

### Harvesting site

*L. leucocephala* leaves were harvested from Mpumalanga Province, South Africa. The harvesting site has a flat landscape with sandy to loamy soil type. The average mean temperature ranges from 12 in winter to 33 °C in summer times. The annual mean rainfall ranges between 400 and 800 mm, with frost that normally occurs during winter season. *L. leucocephala* leaves were simultaneously harvested at two stages of maturity (tender and mature leaves) and stored separately.

### Bulk leaf samples

*L. leucocephala* leaves were simultaneously harvested at two stages of maturity (tender and mature leaves) and stored separately. The leaves were harvested green, air-dried in a well-ventilated laboratory to constant weight and milled through a 1-mm sieve into a powder using a hammer mill before being subjected to chemical analyses. A 50:50 ratio of both tender and mature leaves of *L. leucocephala* leaf meal (LLM) bulk sample was used in the digestibility trial. The study was approved by university ethic committee (Ethics Number NWU 00242–18-A5).

### Diet formulation

Four diets were formulated by supplementing a commercial broiler finisher diets with graded levels of 0, 25, 50 and 75 g/kg of air-dried and milled *L. leucocephala* leaf meal (LLM). The chemical composition and formulation of LLM trial diets are stated in Table 1. The experimental diet formulation was done at a commercial feed manufacturing company, Simple Grower (Pretoria). These experimental diets were formulated to be iso-nitrogenous.

**Table 1:** Gross composition of *Leucaena leucocephala* leaf meal (LLM) diet–based experiment

Dietary treatment %				
	Control	LLM2.5	LLM5.0	LLM7.5
Ingredients				
Yellow maize, coarse	60	60	60	60
Wheat bran	12	12	10	10
*L. leucocephala*	0	2.5	5	7.5
Soybean meal	13	10.52	9.05	6.56
Sunflower oilcake	9.7	9.75	10	10
Limestone powder, fine	2	2	3.2	3.2
MCP/mono Cal KK	1	1	1	1
Salt, fine	0.5	0.5	0.5	0.5
Bicarbonate	0.17	0.24	0.24	0.24
Lysine	1.22	1.04	0.71	0.7
Choline powder	0.05	0.05	0.05	0.05
Methionine	0.36	0.35	0.2	0.2
Premix (%)	0.05	0.05	0.05	0.05
Total (%)	100	100	100	100
Nutrient composition (%) analysed				
Dry matter (%)	90.25	90.81	90.39	90.93
Ash (%)	5.35	6.61	6.11	6.37
Crude protein (%)	19.53	19.50	19.05	19.44
Fats (%)	3.40	5.66	5.10	4.49
ADF (%)	10.25	10.57	10.92	11.17
NDF (%)	20.11	20.67	21.45	21.29
ADL (%)	2.74	3.59	3.77	4.58
Cellulose (%)	7.51	6.98	7.15	6.59
Hemicellulose (%)	9.86	10.10	10.53	10.11

*%*, percentage; *ADF*, acid detergent fibre; *NDF*, neutral detergent fibre; *ADL*, acid detergent lignin; *LLM 2.5*, 2.5 *L. leucocephala* leaf meal inclusion; *LLM 5.0*, 5.0 *L. leucocephala* leaf meal inclusion; *LLM 7.5*, 7.5 *L. leucocephala* leaf meal inclusion

### Chemical analyses

The chemical analyses of each plant and bulk samples of leaf were carried out in the Animal Nutrition Laboratory at the North-West University Experimental Farm (Molelwane). Moisture and dry matter material contents were determined by weighing sample in crucible and drying in an oven over night at 105 °C to reach constant weight (AOAC 2005). The ash content was determined by ashing plant materials at 550 °C for 6 h in a muffle furnace. Total nitrogen content was analysed using the standard macro-Kjeldahl method (AOAC 2005), and it was converted to crude protein through multiplying percentage N content by 6.25. The neutral and acid detergent fibres (NDF and ADF) were assessed by refluxing 0.45 g samples with neutral detergent and acid detergent solutions, respectively, according to Van Soest et al. (1991). Heat stable ἂ-amylase was utilized for analyses of NDF with the exclusion of sodium sulphite. The chemical composition values were used to predict chemical estimates (DMDigest, TDN, DE, ME) of *L. leucocephala* leaves under different growth stages. The formula used to predict total digestible nutrients (TND) was 82.38 – (0.7515 × ADF) as described by Bath and Marble (1989). The formula for dry matter digestibility was DMDigest% = 88.9 – (0.779 ×  % ADF). Dry matter digestibility values were used to estimate digestible energy (DE, kcal/kg) using the regression equation reported by Fonnesbeck et al. (1984), DE (Mcal/kg) = 0.27 + 0.0428 (DMDigest%). DE values were converted to ME using the formula reported by Khalil et al. (1986), ME (Mcal/kg) = 0.821 × DE (Mcal/kg). To assay for soluble condensed tannins (SCT), the aqueous acetone leaf extract (0.5 ml) was utilized by means of modified butanol-HCl reagent (95:5 *v*/v) (Porter et al. 1986). The utilization of a spectrophotometer (T60 UV-Visible Spectrophotometer, PG Instruments) of absorbance was observed at 550 nm wavelength. The mineral content of calcium (Ca), magnesium (Mg), potassium (K), sodium (Na), iron (Fe), zinc (Zn), manganese (Mn) and copper (Cu) of *L. leucocephala* leaves was determined, using the atomic ICP spectrophotometer (AAS-Buck 205) according to the procedures provide by the Agri-Laboratory Association of Southern Africa (AgriLASA 1998).

### Nutrient digestibility

For the digestibility trial, twelve Potchefstroom Koekoek and twelve Black Australorp breeds (twenty-four chickens) were used. A factorial experiment in a completely randomized design (SAS 2010) was used for the trial (two breeds × four diets) with each treatment replicated three times. At the end of the experiment, the chickens were 90 days old when apparent nutrient digestibility was measured. Chickens (3 per treatment) were randomly and individually placed in metabolic pens for estimation of apparent digestibility. A 5-day acclimatization period was allowed prior to a 7-day collection period. Each replicate for collected excreta was kept at −15 °C pending proximate analyses throughout the collection period to preserve the nutrient available on faeces (Gollcher et al. 2010). The feed offered and leftovers were weighed. The apparent digestibility values of dry matter (DM), crude protein (CP), neutral detergent fibre (NDF) and acid detergent fibre (ADF) were measured according to Mcdonald et al. (2005):

$$\mathrm{Apparent}\ \mathrm{digestilbilty}=\frac{\mathrm{Nutrient}\ \mathrm{intake}-\mathrm{faecal}\ \mathrm{nutrient}}{\mathrm{Nutrient}\ \mathrm{intake}}\times 100$$

### Mimosine content

A mixture of sodium nitrates and SAM solutions were added together and permissible to stand for 20 min for diazotization of SAM to occur (DSAM solution). A corresponding liquor of mimosine working standard solutions was also prepared. Test tube series of 0.005–15 g/ml were transferred in duplicate and volume. Each tube was adjusted to 0.5 M HCl. The control tubes were added with 0.5 M HCl. A 1 ml solution of sodium carbonate and 2 ml solution of DZSAM were added. A total of 1 ml of NEDA solution was added together, permissible to stand in a room temperature for 10 min, and then adjusted volume to 6 ml water level. A mixture of solution and colour was developed for 10 min at room temperature. The absorbance values were recorded at 539 nm, and the calibration curve was erected by plotting the absorbance (absorbance, Ao), when absence of mimosine was added absorbance.

### Statistical analysis

The chemical composition data of the leaves was analysed using one-way ANOVA (SAS 2010). The general linear model employed was:

$${Y}_{ij k}=\mu +{S}_i+{E}_{ij}$$

where *Y*_*ijk*_ is the observation of the dependent variable *ijk* (chemical composition of the leaves), *μ* is the fixed effect of population mean for the variable, *S*_*i*_ is the stage of growth of leaves and *E*_*ij*_ is the random error associated with observation *ij* assumed to be normally and independently distributed.

A two-way ANOVA was used to analyse data on the effect of chicken breed, diet and chicken breed × diet interaction on apparent digestibility data. The general linear model (GLM) procedures of SAS (2010) software were employed in this statistical analysis. The linear model employed was:

$${Y}_{ij k}=\mu +{S}_i+{D}_j+{\left(S\times D\right)}_{ij}+{E}_{ij k}$$

where *Y*_*ijk*_ = observation of the dependent variable *ijk*, *μ* = fixed effect of population mean for the variable, *S*_*i*_ = effect of chicken breed (*i* = 2; Potchefstroom Koekoek and Black Australorp), *D*_*j*_ = effect of diet (*j* = 4; LLM0, LLM2.5, LLM5.0 and LLM7.5), (*S* × *D*)_*ij*_ = effect of interaction between strain at level *i* and diet at level *j* and *E*_*ijk*_ = random error associated with observation *ijk* assumed to be normally and independently distributed.

The significance level was set at *P* < 0.05. If the significant variation was detected on chemical components, multiple comparisons of treatment means were carried out using the probability of difference (pdiff) option of the general linear model (GLM) procedures of SAS.

## Results

### Tender and mature leaves

The nutritional composition of *L. leucocephala* leaf meal collected at two maturity stages is presented in Table 2. The obtained results revealed that tender and mature leaves had significantly (*P* < 0.05) similar DM, ash, ADF, NDF, ADL, ether extract, cellulose and hemicellulose. However, there were significantly higher (*P* < 0.05) concentrations of crude protein, mimosine and chemical estimates predicted on tender leaves when compared to mature leaves.

**Table 2:** Nutrient composition (g/kg, unless otherwise stated) and mimosine content of tender and mature leaves of *Leucaena leucocephala* leaves

Parameters	Tender leaves	Mature leaves	SE	*P* value
Dry matter (g/kg)	923.68	920.26	1.443	0.168
Ash (g/kg DM)	84.99	87.42	2.637	0.549
Acid detergent fibre (g/kg DM)	178.81	179.70	3.308	0.859
Neutral detergent fibre (g/kg DM)	234.36	254.44	12.391	0.392
Acid detergent lignin (g/kg DM)	192.41	196.20	4.335	0.569
Crude protein g/kg DM	275.10^a^	255.37^b^	1.278	0.001
Ether extract (g/kg DM)	40.46^a^	23.60^b^	8.212	0.001
Cellulose (g/kg DM)	172.40	172.90	3.332	0.917
Hemicellulose (g/kg DM)	78.30	58.26	11.426	0.335
Condensed tannins (g/kg DM)	24.68^b^	35.55^a^	0.564	0.003
Dry matter digestibility (% DM)	73.91^a^	73.59^b^	0.018	0.008
Total digestible nutrients (% DM)	67.91^a^	67.61^b^	0.018	0.008
Digestible energy (Mcal/kg)	3.43^a^	3.42^b^	0.000	0.008
Metabolisable energy (Mcal/kg)	2.82^a^	2.81^b^	0.000	0.008
Mimosine (g/kgDM)	46.1^a^	25.5^b^	0.577	0.003

^a,b^Means within rows with different superscripts differ significantly (*P* < 0.05). *SE*, standard error; *P*, probability

In Table 3, tender leaf concentrations were significantly higher (*P* < 0.05) in calcium (1.87 g/kg DM), magnesium (3.20 /kg DM), potassium (7.92 g/kg DM) and sodium (2.76 g/kg DM). Iron (347.76 mg/100 g) and zinc (398.22 mg/100 g) contents for mature leaves were higher compared to tender leaves. There were no significant differences detected in manganese concentration in leaf materials.

**Table 3:** Mineral content of tender and mature *Leucaena leucocephala* leaves

Parameters	Tender leaves	Mature leaves	SE	*P* value
Calcium (%)	1.87^a^	1.76^b^	0.041	0.001
Magnesium (%)	3.20^a^	2.65^b^	0.058	0.002
Potassium (%)	7.92^a^	4.12^b^	0.057	0.002
Sodium (%)	2.76^a^	2.53^b^	0.056	0.001
Iron (mg/100 g)	312.60^b^	347.76^a^	4.103	0.002
Zinc (mg/100 g)	250.94^b^	398.22^a^	0.577	0.002
Manganese (mg/100 g)	330.10	342.56	4.103	0.372
Copper (g/100 g)	394.07^b^	484.79^a^	4.082	0.001

^a,b^Means within rows with different superscripts differ significantly (*P* < 0.05). *SE*, standard error; *P*, probability

**Fig. 1:**
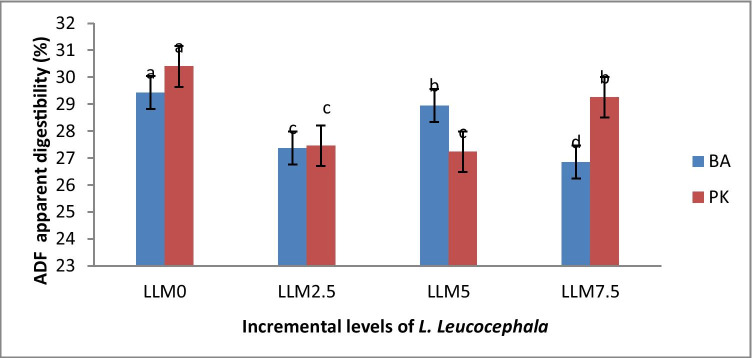
Effect of dietary *Leucaena leucocephala* leaves meal supplementation level (%) on apparent digestibility of acid detergent fibre (ADF) in 12-week-old Potchefstroom Koekoek (PK) and Black Australorp (BA) chicken breeds

**Fig. 2:**
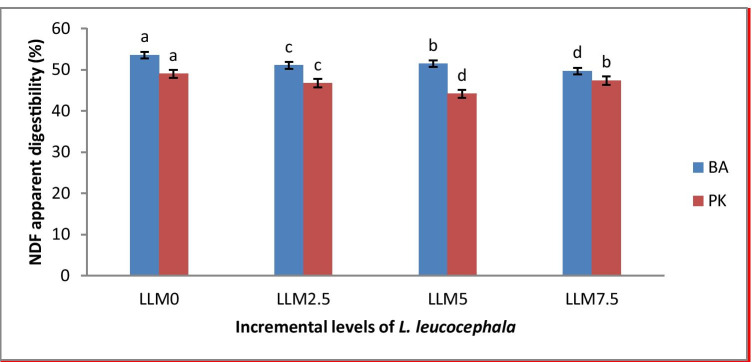
Effect of dietary *Leucaena leucocephala* leaves meal supplementation level (%) on apparent digestibility of neutral detergent fibre (NDF) in 12-week-old Potchefstroom Koekoek (PK) and Black Australorp (BA) chicken breeds

Diet × breed interaction significantly affected apparent digestibility of ADF and NDF (Figs. 1 and 2) and non-significantly on apparent digestibility of dry matter (DM) and crude protein (CP). The Potchefstroom Koekoek revealed the lowest (*P* < 0.05) ADF digestibility (27.23%) in LLM5.0 and the highest ADF digestibility in other diets (Fig. 1). The BA had the lowest ADF digestibility (27.37%) across all incremental levels except in LLM5.0 (Fig. 1). The BA breed showed the highest (*P* < 0.05) NDF digestibility in all diets with the PK breed being the lowest (Fig. 2).

There was higher feed intake in chickens fed 7.5%LLM as compared to chickens fed the control diet (Table 4). The NDF digestibility was similar (*P* > 0.05) for the diets of LLM2.5 and LLM7.5. The digestibility of CP decreased (*P* < 0.05) with increased dietary LLM which was evident with diet LLM7.5. However, the digestibility of ADF, NDF and CP was reduced (*P* < 0.05) with the inclusion of LLM.

**Table 4:** Effects of *Leucaena leucocephala* leaf meal supplementation on feed intake (g) and apparent digestibility (%) of dry matter (DM), acid detergent fibre (ADF), neutral detergent fibre (NDF) and crude protein (CP)

Diet						
Parameters	LLM0	LLM2.5	LLM5.0	LLM7.5	*P* value	SE
Feed intake (g)	1112.50^b^	1123.00^b^	1140.83^ab^	1198.39^a^	0.022	20.43
DM%	89.00	88.53	88.84	88.58	0.99	1.58
ADF%	29.92^a^	27.42^d^	28.09^b^	28.05^c^	0.001	0.29
NDF%	51.26^a^	48.86^b^	47.79^c^	48.47^b^	0.001	0.35
CP%	71.00^a^	53.19^b^	47.47^c^	38.81^d^	0.001	0.17

^abcd^Means within rows with different superscripts are significantly different (*P* < 0.05). *SE*, standard error; *LLM0*, control diet containing 0% of *Leucaena* leaf meal; *LLM2.5*, diet containing 2.5% of *Leucaena* leaf meal; *LLM5.0*, diet containing 5% of *Leucaena* leaf meal; *LLM7.5*, diet containing 7.5% *Leucaena* leaf meal; *DM*, dry matter %; *ADF*, acid detergent fibre %; *NDF*, neutral detergent fibre %; *CP*, crude protein %

## Discussion

### Nutritional composition

The maturity stage and the growing conditions of plant have an effect on nutritional value of forage (Msiza et al. 2021; Sebola et al. 2019). The results of the chemical composition of *L. leucocephala* are in agreement with various reports (Dhär et al. 2007; Mohamed et al. 2014; Reyes and Fermin 2003; Leketa et al. 2019; Ayssiwede et al. 2010). The crude protein content in this study was higher than 23% CP of DM reported by Onibi et al. (2008), but lower than the range of 28–29% CP of DM reported by Munguti et al. (2006). These variations in CP contents might be due to differences in agro-climatic conditions and ages of trees and possibly due to different stages of maturity (Ayssiwede et al. 2010). The results imply that tender leaves contain high protein content which will meet the nutrient requirements of indigenous laying hens better than the mature leaves.

Tender leaves had higher protein content and less fibre, thus have greater potential as a feed additive for high poultry performance due to limited ability of chickens to digest fibre-rich diets (Sebola et al. 2019). At both stages of maturity, the average amount of ether extract is estimated on account that leaves are not a principal source of lipids.

The older plants had higher fibre while there was less CP and digestible dry matter. This has been confirmed by the results of Hassen et al. (2007) for the effect of season on nutritive value of five *Indigofera* species (*I. amorphoides*, *Indigofera arrecta*, *I. brevicalyx*, *I. castata* and *Indigofera cryptantha*). The neutral detergent fibre content of both mature and tender leaves in the current study was less than 35% which will make the diet more digestible (Norton 1994). Higher calcium, magnesium and potassium concentrations were obtained in tender leaves compared to matured leaves. For normal growth, muscle activity and skeletal development in poultry, calcium is required. In laying hens, calcium is also vital for egg shell formation. Both tender and mature leaves can be used in indigenous laying hens’ diets as they will add calcium for optimal growth, bone strength and development. The Ca concentration recorded in this study for air-dried *Leucaena* leaves was similar to the results reported by Leketa et al. (2019). Iron was higher in matured leaves, which is an essential factor of myoglobin and haemoglobin for oxygen transportation and cellular development and division (Kozat 2007). The tender leaves had higher concentration of magnesium than mature leaves. Mg is responsible for chemical reactions in the body and intestinal absorption of Zn (Muhammad et al. 2011). The mineral concentration of *Leucaena* leaves will meet the nutrient requirement for the production for growing chickens. Tannins are being efficiently utilized and included in a components diet of poultry for enhancement of animal overall performance and also to control diseases (Suleyman 2017). Dietary tannins are said to reduce feed efficiency and growth rate in chicks (Dei et al. 2007) when fed in large quantities. Generally, the composition of phenolic compounds can be affected by stage of maturity, post-harvest handling, processing and storage (Sreelatha and Padma 2009). Tannin content was higher in matured leaves, which is possible to hinder performance development in chickens as compared to the tender leaves. In poultry, studies have shown the anti-nutritional tannin effects in chicken feeding; they induce productive performances as a result of reducing feed intake and organic matter digestibility, especially the protein component (Garcia et al. 2004; Longstaff and McNab 2007).

The levels of mimosine measured in this study for the leaves were within the range reported by Longstaff and McNab (2007). The mimosine value was high in the tender leaves as compared to the mature leaves. A similar pattern of a decline of mimosine concentration with extended growth for the leaves was observed by Tangendjaja et al. (1986) for *Leucaena* leaves. The authors observed high levels of mimosine (40–50 g/kg dry weight) in young leaves, but the level fell rapidly within 5 weeks to 10 g/kg DM, and at week 10, the level then gradually decreased to about 2 g/kg DM.

However, the levels of mimosine measured in this study for the leaves were relatively low as compared with the results observed by other authors (Tangendjaja et al. 1986; Adeneye 1991). This might be attributed by the use of the dried *Leucaena* forage in this study.

### Nutrient digestibility

Factors that affect digestibility amongst others are enzymes, hydrochloric acid and bile by endogenous secretions. For effective utilization of feed, it must be digested and absorbed by the chickens (Ndelekwute et al. 2018). The possible effect of anti-nutritional factors, for example, mimosine and tannins present in the *L. leucocephala* plant fed to Black Australorp and Potchefstroom Koekoek chicken breeds, may have caused the apparent changes in digestibility (Safwat et al. 2015; Simon 2012). Different chicken breeds exhibited different apparent digestibility coefficients (Sebola et al. 2018). This could be credited to hereditary contrasts in the ability of each breed to utilize high fibre feeds. The results showed that BA breeds have a better tolerance to fibre and anti-nutritional factors in the diet when LLM was included, even up to 7.5%. The current results revealed no significant dietary treatment on DM digestibility suggesting that chicken breeds were not affected by the fibre and anti-nutritional factors present in the LLM. The results are in agreement with Ayssiwede et al. (2010), who reported that diet with or without LLM fed on hens had no significance effect on utilization value of DM digestibility. In contrast, Abou-Elezz et al. (2012) reported that feeding Rhode Island Red hens 0, 5, 10 and 15% LLM had no effect on NDF digestibility. de Oliveira et al. (2014) reported that DM digestibility decreased by adding 10% LLM in the diets and enhance the excretion of DM in faeces by laying hens fed diet with a higher proportion of fibre. The author further explained that the excretion of DM increases because the fibrous fraction is digested in the digestive tract. A decrease in the protein and the fibrous fraction in the diet of indigenous chickens had no significant effect on DM (Arruda et al. 2010). The dry matter digestibility values obtained in the present study were similar to those reported by Abou-Elezz et al. (2012).

Kakengi et al. (2007) reported that including high levels of 10 and 15% of LLM and MOLM in the diets of laying hens resulted in lower CP digestibility. Iheukwumere et al. (2008) reported similar results with broilers where the digestibility values of DM, CP and CF were lower in 10 and 15% cassava leaf supplemental levels than in the control. Safwat et al. (2015) and Simon (2012) reported similar lower apparent digestibility value of crude protein in indigenous laying hens and in rabbits fed LLM. These reports are in agreement with the Nieves et al. (2002) who reported lower DM and CP digestibility values in LLM diets when compared to the control diets. Decrease in feed intake by anti-nutritional compounds in the diet caused decreased digestibility through the ability to bond with proteins and other nutrients (AL-Mamary et al. 2001; Safwat et al. 2015).

The lower crude protein digestibility of the LLM diet agreed with Nieves et al. (2002), who reported a low crude protein digestibility of LLM fed to rabbits when compared to the control diet. Other authors cited that the indigestible protein fraction in the leaf meal may be found in fibrous components (Jensen et al. 1995). The current findings showed that breed and diet interaction did not affect DM and CP digestibility of the selected indigenous laying hens. Apparent digestibility of crude protein in the diet containing *L leucocephala* leaves was higher than that of Cassava leaves (Ayssiwede et al. 2010; Iheukwumere et al. 2008). Commonly, fibre ratios (soluble fibre vs. insoluble fibre) play an important role on digestion rate and the absorption of nutrients (Sebola et al. 2018). Higher fibre digestibility in the control diet indicates that soybean meal fibre is highly digestible compared to LLM fibre. The observed increment of dietary fibre digestibility with increasing the inclusion level of leaf meals in the diets could possibly be due to the unbalance of dietary fibre fractions in terms of the proportions of cellulose, hemicellulose and lignin from the leaf meals and hence deterioration of fibre digestive capacity.

## Conclusion

Significant variation was obtained in the chemical composition of tender and mature *L. leucocephala* leaves. The study concluded that tender leaves of *Leucaena* had a higher crude protein, mineral and mimosine concentration but lower fibre content than mature leaves. The data on digestibility of this alternative protein source shows that incorporation of LLM in the diets of laying hens did not negatively affect the digestibility of dry matter. The data obtained from this study on digestibility coefficients of dietary nutrients states that the inclusion of LLM in the diets of Black Australorp and Potchefstroom Koekoek chicken breeds could be recommended between 5 and 7.5% of the diet. However, there is a need to assess the effect of incorporating LLM in the diet for feed efficiency and egg production and egg size on hen.

## Data Availability

All data analysed during the current study are available from the corresponding author on request.
